# Type 1 diabetes, periodontal health, and a familial history of hyperlipidaemia is associated with oral microbiota in children: a cross-sectional study

**DOI:** 10.1186/s12903-022-02625-0

**Published:** 2023-01-11

**Authors:** Caitlin A. Selway, Emilija D. Jensen, Alexia S. Pena, Gabrielle Smart, Laura S. Weyrich

**Affiliations:** 1 School of Biological Sciences, University of Adelaide, Adelaide, SA Australia; 2 Adelaide Dental School, University of Adelaide, Adelaide, SA Australia; 3 Department of Paediatric Dentistry, Women’s and Children’s Hospital, Adelaide, SA Australia; 4 The University of Adelaide, Robinson Research Institute, Adelaide, SA Australia; 5 Diabetes and Endocrinology Department, Women’s and Children’s Hospital, Adelaide, SA Australia; 6 Department of Anthropology, The Pennsylvania State University, University Park, PA 16802 USA; 7 Huck Institutes of Life Sciences, The Pennsylvania State University, University Park, PA USA

**Keywords:** Type 1 diabetes, Hyperlipidaemia, Oral microbiota, Periodontal health, Pediatrics

## Abstract

**Background:**

Hyperlipidaemia may play a significant role in the interrelationship between type 1 diabetes (T1D) and periodontal disease. A potential mechanism that links these three aspects together is the oral microbiota. We wanted to determine if there is an association between hyperlipidaemia, periodontal disease, and the oral microbiota of children with T1D, as this has not yet been explored.

**Methods:**

In a post-hoc, cross-sectional study using 16S rRNA gene sequencing, we explored links between oral bacterial diversity and composition of gingival swab samples from 72 children with T1D to periodontal risk factors and hyperlipidaemia status of first-degree relatives. While multiple periodontal risk factors were assessed, we used periodontal pocket depth of 3 mm to characterise periodontal risk. As periodontal pocket depth confounded the analysis of familial history of hyperlipidaemia, a multivariate analyses were performed (i.e., no periodontal risk markers in children with or without a family history of hyperlipidaemia were compared to counterparts who did not have periodontal risk markers) to examine linkages between these factors and diversity and composition of the microbiome.

**Results:**

In participants with no periodontitis risk, children with a family history of dyslipidemia had different bacterial diversity and composition compared to those without a familar hisitory. In contrast, such differences did not exist in the children with periodontal risk, whether or not they had a family history of hyperlipidaemia. Co-occurrence networks showed that these differences in children with no periodontists risk were linked to the presence of fewer oral microbial networks, but more microbes linked to mature plaque structures. In contrast, children with periodontal risk markers, regardless of family history of hyperlipidaemia, contained co-occurrence networks that were associated with microbes linked to periodontal disease.

**Conclusions:**

In children diagnosed with T1D, our findings support an association between oral microbiota and two different exposure variables: familial history of hyperlipidaemia and periodontal risk factors.

**Supplementary Information:**

The online version contains supplementary material available at 10.1186/s12903-022-02625-0.

## Background

Hyperlipidaemia is a multifactorial condition that has been linked to social, environmental and genetic factors [1]. In industrialised countries, hyperlipidaemia is a consequence of many factors, including poor diet, smoking, sedentary lifestyle, diabetes, and familial hyperlipidaemia [2]. Additionally, genome wide association studies have demonstrated over 150 human genomic loci are associated with abnormal lipid levels [3]. However, more than half of the variation associated with circulating lipid levels in the blood has been linked to non-genetic factors, including the microbial communities that colonise the body (microbiota) [3]. In mouse models, gut microbiota have been associated with regulation of blood lipids using particular lipoprotein lipase inhibitors, such as fasting-inducing adipose factor (FIAF) [4]. Further, gut microbiota co-produce secondary acids, such as short-chain fatty acids, bile acids, and conjugated linoleic acids, which are used in metabolic pathways, such as regulating lipids [5] and cholesterols [6]. Although there is emerging evidence on the relationship between hyperlipidaemia and gut microbiota, the systemic mechanisms that underpin these interactions require further investigation.

Interactions between hyperlipidaemia and microbiota elsewhere in the body also require further exploration. Relationships between hyperlipidaemia and other microbiota-associated diseases, such as type 1 diabetes (T1D) and periodontitis, have been proposed [7]. In fact, 29–66% of children with T1D have also been shown to have hyperlipidaemia [8], and hyperlipidaemia is a reported risk factor for periodontal disease [9]. Although T1D has a strong genetic inheritance [10], recent mouse and human studies identified additional correlations between gut microbiota and T1D [11]. For example, decreased abundances of *Bifidobacterium* and butyrate-producing bacteria in the gut, as well as lower overall gut microbial diversity, have been observed in adults with T1D compared to healthy individuals [11]. We hypothesised that oral microbiota may play a role in the connection between both diseases, as well as their interactions with other health outcomes. For example, uncontrolled individuals with T1D and hyperlipidaemia have alterations to wound healing [12] and an increased association for developing periodontal disease [7, 12]. Indeed, our previous study showed that glycaemic control and periodontal markers in children with T1D can influence the oral microbiota [13], although the additional interactions with hyperlipidaemia have not yet been explored.

In this post-hoc cross-sectional study, we explored the effect of familial hyperlipidaemia (parent status) on the oral microbiota of children with T1D, who were enrolled in a study to investigate the effects of glycaemic control (HbA1c) and periodontal risk markers on the oral microbiota [13]. We previously confirmed links between periodontal disease characteristics and changes in oral microbiota [13] but now sought to further explore the links between lipids and the oral microbiota of these children. Our goal was to explore the oral microbiota relationship between T1D, periodontal status and a family history hyperlipidaemia in these children.

## Methods

### Study cohort, sample collection and sample preparation

Seventy-six children with T1D were recruited over a twelve month period at the Pediatric Diabetes Clinic at the Women’s and Children’s Hospital, Adelaide, Australia between 2018 and 2019, under ethics approval (Women’s and Children’s Health Network Human Research Ethics Committee; HREC/17/WCHN/165). Informed written consent was obtained by parents/guardians, or from the children themselves if they were 16 years or older. Children between 8 and 18 years of age with previously diagnosed T1D through detectable islet cell autoantibodies were consecutively recruited and underwent dental examination (Table 1). This age range was selected to include children in their mixed and permanent dentitions with at an appropriate level of dental development and compliance levels to allow for thorough periodontal examination. The exclusion criteria included a diagnosis of diabetes other than T1D, inadequate English skills to understand the information sheet, participants with a fever or infection, participants with diabetic ketosis, or those taking antibiotics at the time of dental examination. Parents confirmed a positive blood test and requiring management for those with a family history of hyperlipidaemia.

**Table 1:** Summary of study participant characteristics

Characteristic	Participants (n = 76)	Participants after filtering samples (n = 72)
Age (years)	13.1 ± 2.7	12.9 ± 2.5
Sex (females; n, %)	39 (51.3)	38 (52.7)
BMI (raw)	22.1 ± 0.76	22.1 ± 3.76
BMI (z-score)	0.80 ± 0.76	0.79 ± 0.77
Duration of T1D (years)	5.51 ± 3.81	5.56 ± 3.90
HbA1c (%; median, range)	8.1 (5.8–13.3)	7.95 (5.8–13.3)
Pocket depth ≥ 3 mm (Yes; n, %)	36 (47.4)	34 (47.2)
Plaque index	0.92 ± 0.52	0.92 ± 0.52
Gingival index	0.66 ± 0.43	0.66 ± 0.43
Bleeding on probing (n, %)	65 (85.5)	63 (87.5)
Family history of hyperlipidaemia (n, %)	30 (39.5)*	30 (41.7)

Mean ± SD unless specified; T1D = type 1 diabetes; BMI = body mass index

*Calculation performed on 74 individuals with available metadata

Information on familial, medical, and dental history were provided by the parents, guardians, or participants, which included current home oral hygiene practices (including frequency of brushing, type of toothpaste, and regular flossing; Additional file 1: Table S1). Dental and periodontal examination was performed by a single trained and calibrated dentist. Periodontal risk markers were measured on six teeth (fully erupted first permanent molars in each quadrant, the right maxillary central incisor and left mandibular central incisor), whereby each periodontal parameter was recorded on six points per tooth (mesio-buccal, buccal, disto-buccal, mesio-lingual, lingual, disto-lingual) by the same operator. Periodontal risk markers including plaque index, gingival index, bleeding on probing, and pocket depth, were evaluated and described further in [13]. For this study, pocket depth (PD) was used as the metric to characterise periodontal risk and is defined as no periodontal PD ≥ 3 mm or periodontal PD ≥ 3 mm. To account for periodontal PD being a potential confounding variable, periodontal risk marker status was separated into no periodontal risk markers (no periodontal pockets with depth ≥ 3 mm; individuals = 38) or periodontal risk markers (at least one periodontal pocket with depth ≥ 3 mm; individuals = 34), prior to assessing the association between the oral microbiota and a family history of hyperlipidaemia. This created four groups; (1) no periodontitis without a family history of hyperlipidaemia (n = 19); (2) no periodontitis with a family history of hyperlipidaemia (n = 19); (3) with periodontitis and without a family history of hyperlipidaemia (n = 23); (4) with periodontitis and with a family history of hyperlipidaemia (n = 11). Body mass index (BMI) z-score was calculated using the Children’s Hospital of Philadelphia Research Institute pediatric z-score calculator (https://zscore.research.chop.edu/). Due to these samples being a convenience sample, sample size was dependent on the recruitment of cases over a twelve month period.

Gingival (plaque) samples were collected by a practitioner wearing a clean mask and gloves. More specifically, a swab was passed against the gingival margin between the tooth and gingiva from distal to medial on the buccal surface of the lower left first permanent molar. The gingival swab was collected at the time of dental examination and was immediately stored in sterile tubes at − 80 °C.

Samples were transported to a dedicated low-biomass microbiome laboratory at the University of Adelaide for DNA extraction (Qiagen Dneasy Powersoil kit) and 16S ribosomal RNA library preparation (see for [13] for detailed methods). DNA sequencing was completed using a 150 paired-end kit on an Illumina MiSeq at the Australian Cancer Research Foundation, Cancer Genomics Facility (Adelaide, Australia). As part of this process, extraction blank controls (EBCs) and no template controls (NTCs) were also processed to identify background laboratory contamination.

### Bioinformatic, microbial diversity, and statistical analyses

As the data for this study was publicly available, demultiplexed DNA sequences were downloaded from the QIITA repository (Study ID 13235). Data processing followed the same pipeline as [13]. Briefly, in QIIME2 ([14]; v2019.7), sequences were joined using vsearch [15] before undergoing quality assessment and denoising at 250 bp with Deblur [16]. A SEPP insertion tree [17] was created, and sequences were assigned using the SILVA database (v132; 16S rRNA gene 515–806). Using decontam [18], contaminant amplicon sequence variants (ASVs) were identified from EBCs and NTCs (prevalence threshold set to 0.6) and were removed from gingival samples. Samples with low sequencing depth (< 5000 sequences) or incomplete metadata, and ASVs with < 11 sequences per ASV were also removed.

Microbial diversity and statistical analyses were performed in QIIME2. At a rarefied depth of 5000 sequences, diversity (alpha diversity) was calculated using observed ASVs (richness) and Faith’s phylogenetic diversity [19] metrics. Significant associations between diversity and categorical metadata were tested using a Kruskal–Wallis [20] pairwise statistical test, whereby FDR adjusted p-values (q-values) were reported. Similarly, differences in bacterial composition diversity were calculated using unweighted UniFrac [21] and Bray–Curtis metrics at a rarefied depth of 5000 sequences. Both PERMANOVA [22] and adonis [23] tests were used to determine statistical differences between groups and the variation that a metadata category contributes to the composition, respectively. We also explored confounding factors between HbA1c, periodontal PD, and a family history of hyperlipidaemia using standard linear regression models in R (version 3.6.1) with the ‘lm’ function. The approached used to explore confounding relationships between these factors was to drop insignificant three- and two-way interactions in a stepwise approach until all significant interactions were identified.

Co-correlations between genera were determined by exporting biom tables from QIIME2 and importing them into MEGAN6 [24]. The taxon chart tool with the co-occurrence function was used, and equal numbers of samples were compared in each instance (n = 11 per group). In MEGAN6, ASV data was collapsed to the genus taxonomic level and visualised as co-occurrence plots using the Pearson correlation test with the following parameters: observed in 80% to 100% of samples and had an edge threshold of 80%.

## Results

### Robustness of oral microbiota signal in gingival swabs

Using decontam, 54 ASVs were identified as contaminants from EBCs and NTCs (Additional file 2: Table S2) and removed from the biological samples. From 76 samples, three individuals had incomplete metadata, while one sample had a low sequencing depth; these four samples were discarded from all downstream analyses. In the remaining 72 samples (Table 1), 905 ASVs were identified from 2,567,677 sequences (range sequences/sample = 5354–94,295; median = 33,920).

A total of 12 phyla were detected from 72 gingival samples. The most abundant phyla (> 2% mean relative abundance) were Proteobacteria (43.3%), Firmicutes (30.7%), Bacteroidetes (9.7%), Actinobacteria (8.3%), and Fusobacteria (6.3%) (Additional file 1: Fig. S1). From 131 total identified genera, 12 genera were highly abundant (> 2% relative abundance): *Haemophilus* (19.2%), *Streptococcus* (16.3%), *Neisseria* (10.3%), *Veillonella* (9.9%), *Aggregatibacter* (5.3%), *Fusobacterium* (3.8%), *Actinobacillus* (3.2%), *Actinomyces* (2.8%), *Leptotrichia* (2.5%), *Corynebacterium* (2.4%), *Prevotella* (2.0%), and *Rothia* (2.0%).

Using linear regression models, HbA1c, periodontal PD, and a family history of hyperlipidaemia were intially independent of one another and not interacting with one another (Additional file 1: Table S3). Subsequently, we removed any insignificant three-way and two-way interactions, and determined that glycated haemoglobin influenced the oral microbiota independently of periodontal PD and a family history of hyperlipidaemia (Additional file 1: Table S4), and that periodontal PD was confounded by a family history of hyperlipidaemia. As a result, two groups were created to account for periodontal risk factors when investigating a family history of hyperlipidaemia: no periodontal risk markers (no periodontal pockets with depth ≥ 3 mm; individuals = 38) or periodontal risk markers (at least one periodontal pocket with depth ≥ 3 mm; individuals = 34) for the following analyses. Overall, there were four groups assessed; (1) no periodontal risk markers without a family history of hyperlipidaemia (n = 19); (2) no periodontal risk markers with a family history of hyperlipidaemia (n = 19); (3) with periodontal risk markers and without a family history of hyperlipidaemia (n = 23); (4) with periodontal risk markers and with a family history of hyperlipidaemia (n = 11).

### Microbiota diversity in children with no periodontal risk markers and a family history of hyperlipidaemia

**Fig. 1:**
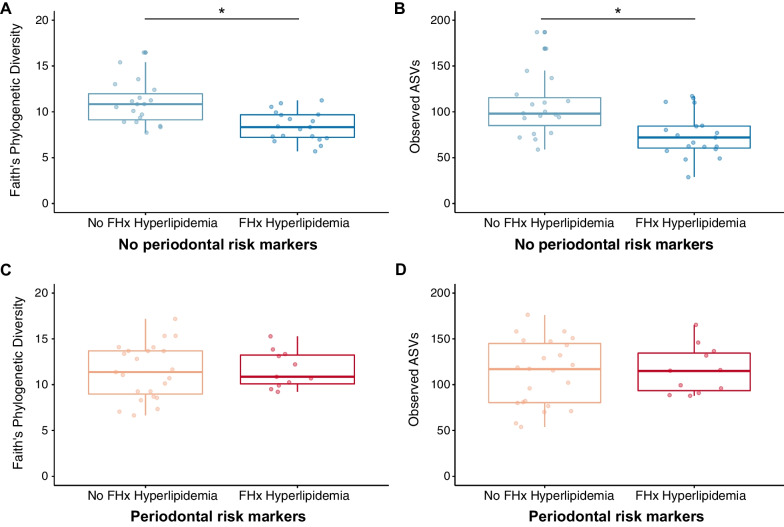
Children with no periodontal risk markers and a family history of hyperlipidaemia have lower oral microbial diversity. Samples were rarefied to 5000 sequences and alpha diversity was measured using faith’s phylogenetic diversity (**A**, **C**) and microbial richness (observed ASVs; **B**, **D**) for children without (blue; **A**, **B**) and with (red; **C**, **D**) periodontal risk markers. Within each analysis, diversity was compared between children without (light) and with (dark) a family history of hyperlipidaemia. *p < 0.05

Children with no periodontal risk markers and a family history of hyperlipidaemia had significantly lower oral microbial diversity compared to those without a family history of hyperlipidaemia (Kruskal–Wallis; phylogenetic: H = 11.174, q = 0.001; richness: H = 8.616, q = 0.003; Fig. 1A, B). However, this was not in the case in children with periodontal risk markers; there were no significant differences between children with or without a family history of hyperlipidaemia (Faith’s PD: H = 0.009, q = 0.927; richness: H = 0.049, q = 0.825; Fig. 1C, D).

### Microbiota composition in children with no periodontal risk markers and a family history of hyperlipidaemia

**Fig. 2:**
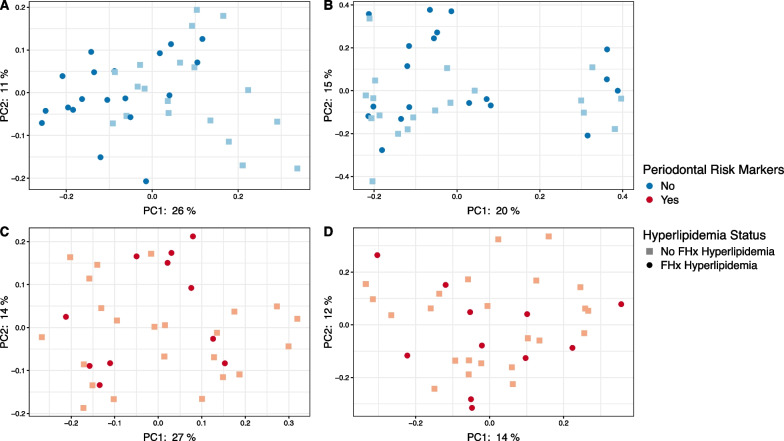
Children with no periodontal risk markers and a family history of hyperlipidaemia have a distinctly phylogenetic microbial composition. PCoA plots were generated to compare compositional differences using unweighted UniFrac (**A**, **C**) and Bray–Curtis metrics (**B**, **D**). Children were separated based on having no (blue; **A**, **B**) or with (red; **C**, **D**) periodontal risk markers before comparing children without (light square) and with (dark circle) a family history of hyperlipidaemia. The principal coordinates analysis axes (PC) explain the total amount of variation, with the first two axes (i.e., x axis = PC1 and y axis PC2) capturing the largest amount of variation in the data

We measured compositional variation using unweighted UniFrac and found a significant difference in phylogenetic composition between children with no periodontal risk markers with or without a family history of hyperlipidaemia (unweighted UniFrac; PERMANOVA: pseudo-F = 4.281, q = 0.001; adonis: R2 = 0.117, q = 0.02; Fig. 2A). However, there were no significant differences for weighted non-phylogenetic composition between these groups (Bray–Curtis: pseudo-F = 1.164, q = 0.265; Fig. 2B). In children with high-risk periodontal markers, we saw no compositional differences between children with or without a family history of hyperlipidaemia (PERMANOVA; unweighted UniFrac: pseudo-F = 0.743, q = 0.725; Bray–Curtis: pseudo-F = 0.948, q = 0.511; Fig. 2C, D).

### Taxa in children with no periodontal risk markers and a family history of hyperlipidaemia

Using ANCOM, a *Prevotella* ASV was found to be significantly more abundant in children with no periodontal risk markers and a family history of hyperlipidaemia compared to children without (W = 202). As only one ASV was significantly more abundant, we looked for the presence of any unique ASVs in children with no periodontal risk markers and a family history children compared to those with no family history of hyperlipidaemia. There were more unique ASVs observed in children without a family history of hyperlipidaemia (412 unique ASVs; n = 19 samples) in comparison to children with a family history of hyperlipidaemia (84 unique ASVs; n = 18 samples).

### Microbial network differences according to the presence of markers of periodontal disease in children with T1D and family history of hyperlipidaemia

Using the Pearson metric, we identified distinct networks of co-occurring genera between children with no periodontal risk markers and a family history of hyperlipidaemia or children without a family history. In children with no periodontal risk markers and without a family history of hyperlipidaemia, four networks were observed (Additional file 1: Fig. S2A), simplified as: (1.) *Campylobacter* and *Fusobacterium*; (2.) *Rothia* and *Corynebacterium*; (3.) *Alloprevotella*, *Porphyromonas*, and *Capnocytophaga*; and (4.) *Streptococcus*, *Gemella*, and *Bergeyella*. In children with no periodontal risk markers and a family history of hyperlipidaemia, there were only two co-occurrence networks: (1.) *Corynebacterium* and *Capnocytophaga*; and (2.) *Fusobacterium* and *Leptotrichia* (Additional file 1: Fig. S2B).

In children with periodontal markers and no family history of hyperlipidaemia, a single network of six species commonly associated with periodontal disease (*Prevotella*, *Porphyromonas*, *Fusobacterium*, *Campylobacter*, *Capnocytophaga*, and *Leptotrichia*) was detected (Additional file 1: Fig. S2C). However, children with periodontal risk markers and a family history of hyperlipidaemia maintained four distinct networks: (1.) *Actinomyces* and *Kingella*; (2.) *Cardiobacterium* and *Bergeyella*; (3.) *Rothia* and *Gemella*; and (4.) *Fusobacterium*, *Leptotrichia*, *Corynebacterium*, and *Prevotella* (Additional file 1: Fig. S2D).

## Discussion

Hyperlipidaemia is a multifactorial disease that may provide a link between T1D and periodontal disease. In this study, the relationship between hyperlipidaemia and periodontal pocket depth in children with T1D was explored using family history of hyperlipidaemia in first degree relatives as a proxy for abnormal lipid profile in their child, given genetic and environmental factors similarities between parents and children. Lower microbial diversity and changes to microbial composition were correlated with a family history of hyperlipidaemia in children with no periodontal risk markers. However, this was not observed for children with periodontal markers, and instead, increased abundance of a *Prevotella* ASV and fewer unique ASVs were observed in this group. In children with no periodontal risk markers and a family history of hyperlipidaemia, networks likely associated with more mature plaque structures were observed. Such microbial differences may indicate that either genetic and/or environmental factors related to a family history of hyperlipidaemia are associated with the oral microbiota in children with T1D.

Children exhibiting no periodontal risk markers with a family history of hyperlipidaemia had lower oral microbial diversity and a phylogenetic compositional shift. However, no significant difference in diversity or composition were observed between children with or without a family history of hyperlipidaemia when children had a pocket depth ≥ 3 mm. We hypothesise that early periodontal markers have a masking effect on the oral microbiota, such that effects of hyperlipidaemia cannot be observed; however, further studies would be required to test this hypothesis. Additionally, children with no periodontal risk markers and a family history of hyperlipidaemia had increased levels of a *Prevotella* ASV, which is a similar observation to individuals with chronic [25] or systemic diseases, such as gout [26]. It was also observed that networks of microbial species were altered in individual’s with periodontal markers in agreeance with current literature [27, 28]. Interestingly, two bacterial networks observed in children with no periodontal markers and with a family history of hyperlipidaemia are consistent with the formation of mature plaque structures [29], which may eventually contribute to periodontal disease. However, networks in children with no periodontal markers and no family history of hyperlipidaemia. The observations from this research suggest that the microbial networks that underpin periodontal disease development in children with a family history of hyperlipidaemia may be in fact be unique compared to children without this familial history.

Most available periodontal studies have also suggested that gingival inflammation is also likely linked to the outgrowth of *Porphyromonas gingivalis* species during the development of periodontal disease in adults [30]. In periodontal disease, inflammation is also typically preceded by the development of large dental plaque structures. This microbial network analysis in children with periodontal risk markers revealed that *Porphyromonas* was not involved in the main networks of microbes in children with a family history of hyperlipidaemia. However, *Prevotella*—only ASV with a significant association with a family history of hyperlipidaemia children and was found in a microbial network with *Fusobacterium*, *Leptotrichia*, and *Corynebacterium* species—was identified in the networks of children with high-risk periodontal markers and a family history of hyperlipidaemia. It is possible that non-*Porphyromonas* species, such as *Prevotella* species, can significantly contribute to the development of periodontal disease in these children, or that the microbial ecosystem in these children is disrupted in unique ways with putative pathogens that can increase in abundance during periodontal disease and stimulate an immune response [31].

A relationship between hyperlipidaemia and periodontal disease has been described and how this affects the oral microbiota has not been previously explored. Both mouse and human studies have shown that high fat diets and high blood lipids are associated with periodontitis [7]. Increases in blood lipids can elevate proinflammatory cytokines [7], which can then reside in the gingival crevicular fluid and promote inflammation of the gingiva. This process of gingival inflammation and the infiltration of proinflammatory cytokines is indicative of periodontal disease. Alternatively, this process of increased inflammatory cytokines may be bi-directional with the trigger of inflammatory state being a result of periodontal disease [7]. To better understand the relationship between hyperlipidaemia and periodontal markers, further evaluation involving taxonomic and functional differences of oral microbiota in children with and without a family history of hyperlipidaemia and with or with no periodontal risk markers should be considered for a larger study.

This study was limited by the three factors: (1) the status of hyperlipidaemia from first-degree relatives (i.e., parent/s) was used as an indicator of potentially abnormal lipids in children, rather than direct lipid assessment in the children; (2) a full-mouth periodontal examination was not carried out; and (3) gingival swabs were collected rather than plaque samples. Both serum and salivary lipids were not collected for this study as this was not part of the design for the original study. As such, family history of hyperlipidaemia was used as an indicator of lipids for the children. Our assumption is derived from the likelihood that children are exposed to similar lifestyle habits, e.g., diet and exercise patterns, and they share the same genetic make-up as their parents [32–34]. In addition, previous work suggests that between 29 and 66% of children with T1D also had hyperlipidaemia [8, 35], suggesting that our assumption is not unreasonable. As our study group consisted of children, we only assessed six teeth (fully erupted first permanent molars and one upper and one lower central incisor) for periodontal disease risk markers, as these teeth were present and fully erupted in all children. This partial periodontal assessment provided a broad indication of periodontal health, rather than a comprehensive examination as required by the 2018 WHO definition of periodontal health. Likewise, for collection we swabbed the gingival margin between the tooth and gingival of the lower left first permanent molar for consistency of microbiota microenvironment between children, rather than collecting plaque samples themselves. While the swab sample is reflective of plaque, we appreciate that it may have mixed the oral geographic signature maintained in plaque; however, swab samples were well tolerated by children as part of a rigorous full dental assessment.

As the relationship between diabetes, hyperlipidaemia, and periodontal disease ranges from the gut to the mouth and includes the circulatory system, it is important to think of the body as a whole system, rather than focusing on one body site [36]. For example, diabetes studies conventionally focus on gut microbiota, while periodontal studies predominantly focus on the oral microbiota. While a few emerging studies have now begun to examine links between oral and gut microbiota in periodontal disease [37], future studies should focus on the mechanistic interactions between increased circulating glycated haemoglobin, lipids, and proinflammatory molecules. As we gain more knowledge of T1D and potential treatment options evolve, it is important to consider the entire ecosystem of both the mouth and gut, which includes microbiota and the effects of circulating metabolites and cytokines.

## Supplementary Information

**Additional file 1.**  Supplementary tables and figures.

**Additional file 2.** List of contaminant ASVs identified from laboratory controls. 

## Data Availability

The raw dataset is available through the European Nucleotide Archive https://www.ebi.ac.uk/ena/browser/view/PRJEB39172.
