# Supplementary material for: Type 1 diabetes, periodontal health, and a familial history of hyperlipidaemia is associated with oral microbiota in children: a cross-sectional study
Source: BMC Oral Health. 2023 Jan 11;23:15. doi: 10.1186/s12903-022-02625-0 (PMC9832783; doi:10.1186/s12903-022-02625-0)
Supplement: Supplementary file 1 — Additional file 1. Supplementary tables and figures. [file 12903_2022_2625_MOESM1_ESM.pdf]

Table S1: Oral hygiene practices of study participants

Characteristic	Participants after filtering samples (n=72)
Self-reported brushing frequency (n, %)	
No tooth brushing	2 (2.8)
Brushes Sporadically	14 (19.4)
Brushes once a day	29 (20.3)
Brushes twice a day	24 (33.3)
Toothpaste with fluoride (n, %)	70 (97.2)
Regular flossing (n, %)	6 (8.3)

Table S2: Contaminant ASVs identified in extraction blank and negative template controls.

Table S3: No interaction between periodontal pocket depth, glycated haemoglobin, and a family history of hyperlipidaemia.

	Sum of Squares	F value	Pr (>F)
Periodontal PD	6.5486	1.2083	0.27578
HbA1c	25.6292	4.7290	0.03336*
FHx Hyperlipidaemia	12.6063	2.3261	0.13215
Periodontal PD:HbA1c	6.7710	1.2494	0.26785
Periodontal PD:FHx Hyperlipidaemia	3.3294	0.6143	0.43606
HbA1c:FHx Hyperlipidaemia	21.7358	4.0106	0.04946*
Periodontal PD:HbA1c:FHx Hyperlipidaemia	6.9440	1.2813	0.26189

PD = pocket depth; FHx = family history of hyperlipidemia; HbA1c = glycated hemoglobin; * 0.05 > p > 0.01.

Table S4: Periodontal pocket depth and a family history of hyperlipidaemia dependently affect the oral microbiota.

	Sum of Squares	F value	Pr (>F)
Periodontal PD	0.121	0.0220	0.882476
HbA1c	25.386	4.6078	0.035452 *
FHx Hyperlipidaemia	56.926	10.3324	0.002012 **
Periodontal PD:FHx Hyperlipidaemia	39.573	7.1828	0.009255 **

PD = pocket depth; FHx = family history of hyperlipidaemia; HbA1c = glycated hemoglobin; * 0.05 > p > 0.01, ** p < 0.01.

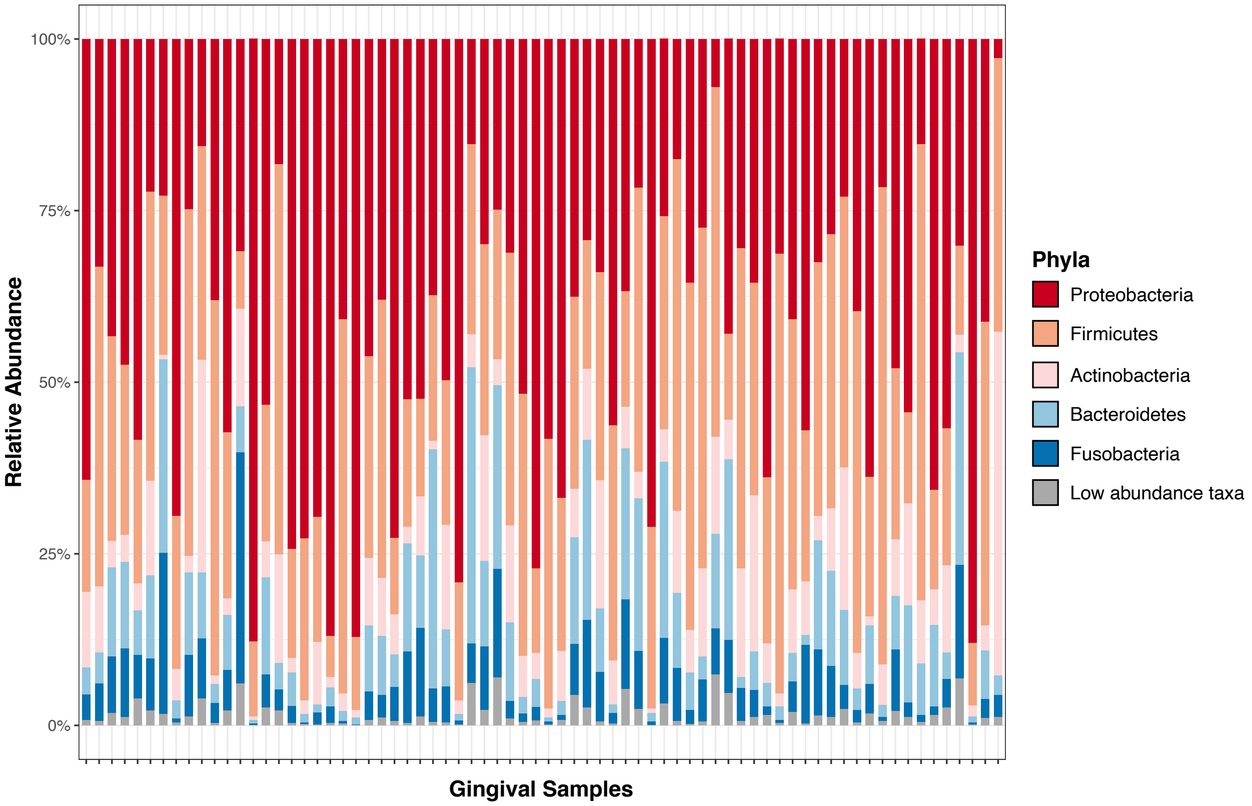

Figure S1: The relative abundance of gingival samples at the phyla taxonomic level. Five dominant phyla (>2% relative abundance) were observed across all gingival samples from children with type 1 diabetes.

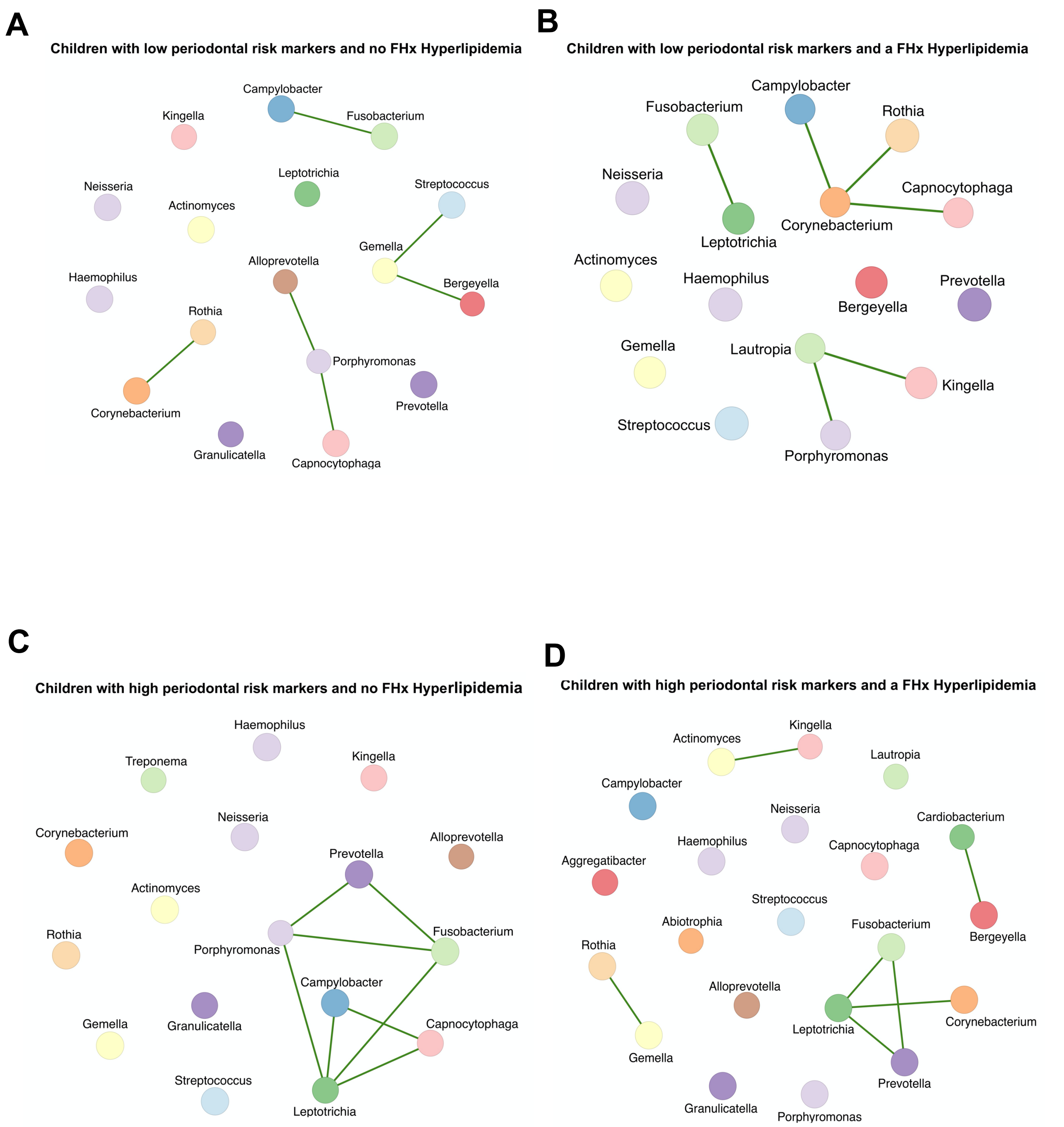

Figure S2: Fewer co-occurrence networks were observed in children no periodontal risk markers and a family history of hyperlipidaemia. Co-occurrence plots were generated using the Pearson co-efficient, to compare differences in hyperlipidaemia and periodontal status. Networks between children with no periodontal risk markers and those without (A) or with (B) a family history of hyperlipidaemia were distinct, with fewer networks observed in periodontally healthy children with a family history of hyperlipidaemia. In children with high-risk periodontal markers, frequently observed periodontal pathogens were shown to co-occur in both children without (C) and (D) with a family history of hyperlipidaemia.
